# Current Understanding of What Infants See

**DOI:** 10.1007/s40135-014-0056-2

**Published:** 2014-10-25

**Authors:** Lea Hyvärinen, Renate Walthes, Namita Jacob, Kay Nottingham Chaplin, Mercè Leonhardt

**Affiliations:** 1 Faculty of Rehabilitation Sciences, TU Dortmund, August-Schmidt-Straße 4, 44227 Dortmund, Germany; 2 Present Address: 644 Whitetail Drive, Lewisberry, PA 17339 USA; 3 Faculty of Rehabilitation Sciences, TU Dortmund University, 44221 Dortmund, Germany; 4 Perkins International, Watertown, MA USA; 5 Chetana Trust, 15 Arunachalam Road, Kotturpuram, Chennai, 600085 India; 6 National Center for Children’s Vision and Eye Health at Prevent Blindness, Chicago, USA; 7 Vision and Eye Health Initiatives, Good-Lite, 42 East Street, Westover, WV 26501 USA; 8 Early Intervention Ramon Marti Bonet Foundation against blindness, Barcelona, Spain; 9 ICR Catalan Institute of Retina, 08172 Barcelona, Spain

**Keywords:** Infancy, Vision development, Early communication, Early intervention, Face recognition, Autism

## Abstract

The current understanding of what infants see varies greatly among healthcare and education specialists. Even among ophthalmologists and pediatric neurologists in charge of clinical examinations of infants, opinions vary on what infants perceive, recognize, and use for communication and learning. It is, therefore, of interest to review publications from several specialties to learn whether new information is available on the development of visual functions and use of vision. Ten percent of total publications on this subject are reviewed here based on the usefulness of their content for improving early diagnosis and intervention of vision disorders in infants.

**Electronic supplementary material:**

The online version of this article (doi:10.1007/s40135-014-0056-2) contains supplementary material, which is available to authorized users.

## Introduction

While collecting material for this review article, it quickly became apparent that the number of publications originally included was overwhelming. Publications included numerous traditional reports based on observations and interpretations of infants’ behaviors. A great number of investigations were based on indirect measurements of brain functions using event-related potential (ERP), EEG techniques, and functional Near Infrared Spectroscopy (fNIRS) and comparing the findings in infants with those from young adults in whom the whole brain was searched for areas of coherent magnetoencephalographic (MEG) activity [1].

Most readers of this article are likely to search for something to use in clinical work; therefore, reports of observational studies are organized around the visual milestones so that the findings can be used to design tests and observation situations. Reports focus on the 1st year of life because this is the most important year in the development of vision, as well as the least well-understood age in clinical work.

An overview of the basic research investigations on functions of the visual brain [2•] is described by Vanderwert and Nelson in 2014.

Deviations from typical visual development are often present at, or soon after birth in infants with normal-looking eyes. The groups of infants at risk with vision impairment are well known and include small, prematurely born infants; brain damage during or soon after birth; infections; epilepsy; and all disorders causing hypotonia, especially Down syndrome. These infants should be carefully observed for symptoms and signs of atypical visual functioning due to disorders in the eyes, in the visual pathways, and/or in the visual brain. This infant group has other more discernible disorders that require intensive care; thus, vision is often not considered in early intervention even when the infants receive other clinical and rehabilitation services.

Eye disorders of healthy infants are detected early during the 1st year whereas atypical use of vision is often met with a “wait-and-see” response. Consequently, clinical diagnostic work of atypical visual functioning of these otherwise healthy infants with normal-looking eyes is delayed because referrals are delayed. Early intervention for infants with impaired vision may not begin at all during the 1st year of life, especially if visual symptoms are interpreted to be “autistic-like.” Because the first 6 months of life are important in the development of infants’ eye contact, early interaction, and development of motor functions, more space has been allotted in this review for the first than the latter half of the 1st year. In order to notice atypical use of vision, typical developmental steps should become better known. They are mentioned in Table 2 (in Additional Information) and the five often overlooked milestones in Table 1.

**Table 1:** The important functional visual milestones related to communication, motor functions, perception and recognition of forms, and awareness of and orientation in space during the 1st year can be easily observed in typically developing infants

1. Vision in the newborn period	1st milestone
2. Eye contact and early interaction	2nd milestone
3. Awareness of hands, motor functions, and anticipation	3rd milestone
4. Recognition of faces, moving, and form perception	4th milestone
5. Matching abstract forms in examination of vision	5th milestone

## Vision in the Newborn Period

Young infants attend and respond to faces of their parents and can copy some facial expressions and tongue movements, as many parents can confirm. A newborn infant’s visual system is tuned to detect social stimuli, such as faces and biological movement. This innate attention to faces has been recorded using hemodynamic response over bilateral posterior temporal cortex [3] in 1–5-day-old infants who were viewing dynamic face stimuli. The hemodynamic response was not recordable when infants viewed movement of other body parts or mechanical movement. This has been documented earlier in infant rhesus monkeys and was interpreted as the first sign of functioning in the mirror neuron system [4].

Body awareness is the foundation on which space awareness is built. It starts to develop based on visual, auditory, tactile, and haptic information at birth and, later, on postural information and experiences of how different body parts move and function. Multisensory integration and the combination of timely synchronized visual and tactile information have demonstrated that from 12 to 103 h of age at the time of testing, human newborns seem to detect intersensory synchrony when related to their own bodies [5]. Arm and hand movements of newborn babies are often interpreted as unintentional. However, in the 1990s [6], arm and hand movements were shown to be purposeful and related to the use of vision (e.g., when the baby could see the hand either directly or in a real-time video image). Infants start learning early on about spatial relationships and how far they can reach with their hands. If this originally normal learning is later disturbed by amblyopia, so that stereopsis and hand movements are not quite typical, it is difficult to know the relationship between these two functions [7].

Newborn infants can recognize physical causality events; for example, they possess an early basic mechanism to compute causality and respond to well-defined spatiotemporal cues present in an event without any prior visual experience of the same kind (*N* = 12) [8]. Newborn infants in this study did not respond if the event parts were temporarily swapped. This ability requires well-organized visual perception, short-term memory, and recognition of the cues during the second presentation. Further, this ability shows that there is a good foundation for further learning [8]. As this test situation is possible in assessing newborn infants, it can be investigated in older infants for measurement of visual brain functioning.

Biological motion point-light tests [9] interest typical newborn infants [10, 11]. Two-day-old babies exposed to a biological motion point-light display and a non-biological motion display (a rotating rigid object) preferentially looked at the biological display. Biological motion could thus be used in testing visual functioning earlier than it is used presently. Perception of biological motion can remain functioning when all other forms of motion perception are severely impaired [12], which shows that it has specific pathways in the brain.

The reviewed material contained few publications on recognition of Kanizsa illusory contours by newborn infants whereas positive responses to a Kanizsa figure in a kinetic display, but not in the static condition, were reported by Valenza and Bulf in 2007 and 2011 [13, 14]. In 2012, one publication reported responses to illusory contours in infants at the age of 4 months [15], and in 2013, two publications reported responses to illusory motion in infants 6–8 months of age, but not at the age of 3 months [16, 17]. These findings show that illusory contours could be developed as a clinical test for early visual processing functions [18] that might shed light on reading problems of children who are slow readers despite having high normal optotype acuity and low grating acuity [19], and on different types of amblyopia.

## Eye Contact and Early Visual Interaction

Looking at faces and responding differently to friendly, smiling, and non-friendly, expressionless faces develop during the 1st and 2nd month [20, 21]. At the age of 6 weeks, a typically developing infant has stable eye contact with his or her parents. If no enjoyable contact occurs by the age of 8 weeks, the infant should be referred to an early intervention service and an ophthalmologist for assessment of vision for early intervention, in addition to the structure of the eyes. Early intervention services for the family should begin without delay and should focus on the important goal of enhancing communication and emotional connections with the infant. Referrals for early intervention are essential to ensure the family receives support and guidance on how to develop and maintain strong communication with their baby already *before* the examinations.

A delay in the development in visual communication was called “*Developmental Emergency”* by Patricia Sonksen in her lectures in the 1980s (published 1997), stressing the need for immediate early intervention [22] to support parents in developing skills to use all sensory and motor cues for communication and interaction and to keep the infant in close bodily contact for comfort and bonding, as in the Kangaroo Care (KC) [23••]. KC was found effective in reducing stress in young infants during the first few months of life and had favorable influences on the development of infants and mothers for at least 10 years. KC could be beneficial after stressful examinations and operations during infancy.

Because infants of blind parents do not have eye contact with their mothers, other communication strategies must be used [24]. These infants learn effective visual, vocal, and auditory communication. For example, they use vocalizations to demand their mother’s attention earlier than typically developing infants.

The recommendation of 8 weeks of age for referral to an ophthalmologist is not always followed, and the family is asked to “wait-and-see” for the visual communication to begin. A prospective, longitudinal study of infants at risk of autism (*N* = 59) revealed that 12 infants who originally had eye contact as if possessing a nascent understanding of mental states showed a slow decline in their visual communication, losing eye contact between ages 2–6 months of age. Later, they did not develop age typical interaction skills [25].

“Autistic-like” behaviors are commonly seen in visually impaired infants with high hyperopia causing such blurred near vision that infants do not learn to accommodate and converge. They see their parents’ eyes as blurred and double. This blurred vision may also be due to modest hyperopia combined with a delay in accommodation development (video #1, Additional materials), which causes severely impaired vision if *near vision correction* is not prescribed. A rare cause of failing eye contact at 2 months of age is face blindness, or prosopagnosia, due to either acquired damage to the face recognition area in the temporal lobe or an even rarer inherited familiar prosopagnosia. In both types, some perception of faces occurs, but the connection to memory functions is weak [26–28]. Infants with face blindness should learn specific communication skills to recognize people by their other typical features, such as hair or jewelry consistently worn close to the face such as a nose ring, earrings, or a necklace, which then becomes the recognized tactile symbol of the mother or caregiver. The fathers’ face can also be explored for specific discrimination details.

At the age of 3 months, the face perception system begins to develop toward having a special interest in human faces, especially in eyes and facial expressions [29, 30]. Typically developing infants and mothers are emotionally close to each other. In a study on social attention or socio-emotional development, the dyadic processes, such as synchrony and bi-directionality, were studied in 133 mothers and their healthy 2-month-old infants. The cohort was created of mothers with high, moderate, or low total score for depression symptoms. During the 3rd month control, interaction between the mothers and infants was videotaped. Results indicated that mothers with high levels of depressive symptoms and their male infants experienced difficult interactions [31]. If an infant turns his/her gaze away from the face of the mother during communication, this could be a warning sign that should be addressed [32].

Facial expressions are low contrast shadows in motion; thus, both contrast sensitivity and motion perception are important for the development of interaction between infants and their caregivers. Global motion sensitivity has been investigated in infants between 3 and 7 months of age and was found to be close to adult function at the age of 3 months *when the test situation was adjusted on the level of contrast sensitivity of each infant* [33••]. In vision research, measurement of basic visual functions, such as contrast sensitivity, is rare, which decreases the value of observations because “improvement” in the observed function could be explained by improvement in contrast sensitivity during the 1st year.

In the investigations reviewed, up to 25 % of subjects were unable to perform in the test situation because of “fuzzy behavior.” Basic clinical examination of the subjects and assessment of their general developmental level were not performed; thus, the reasons for “fuzzy behavior” are unknown. How well the subjects in these studies represent typical infants of their age group remains unclear.

Because fixation is often used as the function observed in psychological investigations, it should be recorded using a standardized test situation when choosing subjects for studies. For example, Robert Fanz standardized the stimulus for fixation as a picture of a face 5 cm in diameter for infants at the age of 3 months [34]. Another important piece of missing information is refraction. Functions such as visual sphere for visual communication, perception and response to figure-in-motion, and responses to gratings are easy and quick test situations [35] that should be used when choosing young infants for psychological investigations.

Young infants are keen observers of faces. The use of head-mounted cameras on infants revealed that between 1 and 3 months of age, infants looked at faces 25 % of the time, primarily female faces (70 %), their own race (96 %), and at adult-age (81 %) [36], which may explain why young infants respond more strongly to pictures of smiling females than males [37, 38].

## Awareness of Hands, Motor Functions, and Anticipation

Vision is an essential component in awareness of hands and in learning how to use them. Hands are our “second set of eyes” and make infants aware of the concrete form, size, weight, and surface quality of objects perceived as abstract visual information. During the 1st month of life, it is possible to observe that some infants look intensively at a hanging toy and then hit it repeatedly with a targeted arm and hand movement (video #2). Such activity gives infants information on how far they can reach with their hands and is one of the cornerstones in learning to understand egocentric small spaces. In 1993, Clifton et al. [39] tested reaching in infants between 5 and 25 weeks of age. Their results indicated that proprioceptive cues, not the sight of the limb, guided the earliest reaching efforts. In 2013, the role of vision for hand movements was evaluated in 5-month-old infants by occluding vision when the infant was reaching for a target [40]. By the age of 5 months, occlusion of vision led to decreased straightness of arm displacement toward the toy as compared to full vision.

Comparing gazing and grasping responses to interesting objects in 4-10-month-old infants, Kanakogi [41] found that infants’ ability to predict the goal of others’ actions was synchronized with the onset of ability to perform that same action [41]. In 2011, Daum [42] made similar observations in a cohort of 6-month-old infants, where action perception and action control were found to be closely related: infants who had developed thumb-opposite grasping showed in their looking behavior more advanced interpretation of the test material than infants who used palmar grasping. Observing infants’ activities in their home environment, we see their hands developing from grasping to exploring.

Vision for hand movements is processed in the parietal/dorsal networks^1^; at the same time, visual information is processed in the temporal/ventral networks for recognition of the objects to be touched or grasped [43, 44]. Functional dissociation in the development of ventral and dorsal pathways has been shown between visually perceived size of real and illusory objects (a visual sensory task) and the adjustment of grasping fingers (a fine motor task) [45]. This dissociation may emerge quite early, although it has not been possible to document it below the age of 5 years [46].

Processing of visual information in the dorsal and ventral networks is usually well synchronized. If visual processing of motion information is not in sync with the movement of the arm and hand, it disturbs performing movements. To avoid this disturbance, the infant/child creates the visual map before reaching and then turns the head away so visual information is not disturbing the movement. If turning of the head in this situation is a socially unwanted behavior, the infant can be taught to close his eyes or look down while reaching. Synchronization may improve if training with slow hand movements is included in the early intervention program.

Motion perception and use of motion parallax in experiencing distances from objects are well developed in many 6-month-old infants. Use of motion parallax was demonstrated by Condry [47] in 2013 in a test situation where the infant was using one eye (the other eye was covered) and was moved in one direction with the same speed as a test object in front of him while a second test object moved with the same speed in the opposite direction. This arrangement makes the speed of the second object appear higher than that of the first object in relation to the infant. Based on motion parallax, this object seems to be closer than the first object and infants preferred to reach for it. When infants used stereovision for estimation of distances, they did not prefer the faster moving object. These results provide the first direct evidence that young infants use spatial information provided by motion parallax to perceive the relative distances of objects and to direct their hand movements.

Motion perception is rarely assessed in clinical examination and is often not mentioned as a function to be evaluated for classification of visual functioning in children, although it is one of the two most important visual functions. The other important function is contrast sensitivity.

## Recognition of Faces, Moving, and Form Perception

Palmar grasp develops to an efficient pincer grasp about 1 year of age. In communication, infants learn to use hands, and later their index finger, to point at objects and activities to share information (declarative pointing) and make adults aware the infant wants something (imperative pointing) [48, 49]. Infants become increasingly skillful in directing their attention and using vocalizations, hand gestures, and body language in social activities [50, 51]. Bilingual infants and infants of deaf parents sometimes develop hand gestures and pointing earlier than typically developing infants. Use of pointing is related to how often pointing occurs during play and communication situations with their family members and caregivers.

Typically developing infants experience their environment from different points of view when lying down; when sitting on the floor, on a lap, in a highchair, or when carried in adult arms; and when they crawl and learn to pull to stand. At the same time, visual communication becomes richer: infants confirm with a brief gaze contact at the adult’s face and eyes that the adult person has noticed and accepted their activity (video #3). In goal-directed actions, the hands and eyes of the adults are coordinated both temporally and spatially. Thus, when infants and parents coordinate their looking behaviors by attending to objects held by either one of them, this eye–hand coupling leads to coordinated shifts in visual attention and to looking at the same objects [52–54].

At the age of 8–10 months, infants begin to recognize their family members by facial features before the family members speak to the infant. Among typically developing infants, some do not develop face recognition and their communication looks unusual because they do not look directly at the eyes of the adults. These infants have congenital, inherited prosopagnosia, face blindness. This behavior is difficult for many caregivers to understand and accept without support and guidance. The deficit in recognition functions may be limited to face recognition (the link to memory is weak or absent). Many features of the face, such as age and gender, may be interpreted normally [55]. In others, holistic processing of information related to the mouth may be normal while processing information related to the eyes is difficult or absent [56]. This difference in information processing resembles changes in face processing during the 1st year [57]. In several families, the deficit can be present in other visual tasks that require holistic integral perception of shape dimensions [58–60].

Inherited prosopagnosia, congenital face blindness, is rare and should be recognized as a familial, non-progressive condition different from face blindness in infants and children with brain damage. The acquired form of face blindness is often connected to problems in several other brain functions, especially to problems in recognition of landmarks. In both forms of face blindness, it is important to explain to the family that the child is NOT autistic and must use blind strategies in visual communication because facial details are not seen normally and memory for facial features is weak.

More mild facial recognition issues have been associated with strabismic amblyopic eyes. [61••]. Most clinicians are accustomed to think about strabismic amblyopia as changes in the early processing of information in the visual cortices, although varying problems have been described in recognition functions. In 2013, Cattaneo et al. [61••] reviewed earlier publications and reported their study on face blindness was limited to face recognition with the amblyopic eye in 10 strabismic amblyopes. Detailed measurements on discrimination of configural details revealed deficits in this function when the amblyopic eye was used. (“Configural details” are distances between eyes and distance between nose and mouth characteristic to each face.) With the dominant eye, the subjects saw the structure of the faces like the normally sighted control subjects [61••].

## Matching Abstract Forms, Getting Ready for Vision Testing

Amblyopia remains the most common vision disorder in young children and theoretically is a preventable disorder if detected early. Currently, treatments begin too late in many countries due to the structure of vision screening. Treatment can be “effective in reducing visual acuity deficits but leaves many amblyopic individuals only partially treated with ocular motor abnormalities, deficient fine motor skills, and risk for recurrent amblyopia” [62]. Recent publications report associations between hyperopia and other vision and refractive error characteristics [63] and variation of anisometropia and amblyopia in cohorts with different ethnicity [64] but none discussed visual functioning. Jost [65] questions the present vision screening strategies and recommends earlier observations, earlier screening, and effective test methods. If prevention and earlier treatment of amblyopia were to become the goals of future care, outcomes would be better and treatment costs for the parents and the public health care system would be lower.

It was surprising to see distinguished journals publishing reports on new visual acuity charts that do not comply with national and international design standards for vision tests [66–69].

When infants are allowed to play with puzzles (video #4 and #5), their form recognition develops early so they can be trained for measurement of visual acuity before they are 30 months old, especially if there are any worries about symmetric development of visual acuity. Puzzles also give an opportunity to observe eye–hand coordination, short-term visual memory, and awareness of directions and distances in the egocentric space.

## Conclusion

The overwhelming number of articles published is a sign of growing interest in the development of vision. Visual abilities of infants in the newborn period and in the first 6 months should become an integral part of monitoring and assessing each infant’s vision development. As primary care providers are educated about infant visual development, more infants can be referred to ophthalmologists and rehabilitation professionals early when the best results can be achieved. Many infants with impaired vision have multiple disorders which bring them to the care of pediatricians and a pediatric neurologist. If these infants are to be identified and referred for the eye care they need, all physicians caring for infants need to be educated about what is typical and what is atypical visual behavior. Prevention of blindness occurs at three levels: primary, secondary, and tertiary. If primary prevention is unsuccessful and the secondary prevention did not result in normal functioning, then early diagnosis and intervention are the tertiary level for prevention of blindness.

## Electronic supplementary material

Below is the link to the electronic supplementary material.

Supplementary material 1 (DOC 29 kb)

## Footnotes

1 Dorsal and ventral pathways have been called “streams,” although the flow in a stream is in one direction, whereas in these large networks, the Top–Down flow is stronger than the flow from the occipital visual cortices toward the higher associative cortical areas.
