# Supplementary material for: Current Understanding of What Infants See
Source: Curr Ophthalmol Rep. 2014 Oct 25;2(4):142–9. doi: 10.1007/s40135-014-0056-2 (PMC4243010; doi:10.1007/s40135-014-0056-2)
Supplement: Supplementary file 1 — Supplementary material 1 (DOC 29 kb) [file 40135_2014_56_MOESM1_ESM.doc]

Table 2. Ages and corresponding behaviours

| **Age (months)** | **Behavior** |
| --- | --- |
| 0–1 | • turns eyes and head to look at light sources  • horizontal eye tracking, tonic focusing |
| 2–3 | • intense eye contact at 6-8 weeks  • vertical and circular tracking  • interested in mobiles  • interested in lip movements |
| 3–6 | • watches own hands  • reaches toward, later grasps hanging objects  • observes toys falling and rolling away  • shifts fixation across mid-line  • visual sphere of attention widens gradually  • very active in visual interaction |
| 7–10 | • notices small bread crumbs, touches them  • adjusts the grasp to the size of the objects  • interested in pictures, also stereo images  • recognizes partially hidden objects  • recognizes family members by facial features |
| 11–12 | • knows places at home  • looks through window and recognizes people  • recognizes pictures, plays hide-and-seek  • can predict adult’s goals of motor actions |
